# Imaging Approaches to Quantifying the Biomechanical Properties of the Human Vitreous In Vivo: A Review

**DOI:** 10.3390/bioengineering13101164

**Published:** 2026-10-06

**Authors:** Taariq Mohammed, Giuliano Scarcelli, Osamah Saeedi

**Affiliations:** 1 Department of Ophthalmology and Visual Sciences, University of Maryland School of Medicine, Baltimore, MD 21201, USA; scarc@umd.edu (G.S.); osaeedi@som.umaryland.edu (O.S.); 2 St. John Center for Translational Engineering and Medicine, University of Maryland, Baltimore, MD 21201, USA; 3 Fischell Department of Bioengineering, University of Maryland, College Park, MD 20742, USA

**Keywords:** ophthalmology, vitreous, vitreoretinal surgery, imaging, biomechanics

## Abstract

The biomechanics of the vitreous humor undergo important changes in both normal aging and various pathologic conditions. While these changes are clinically visible, there are limited objective in vivo measurements of vitreous biomechanics. New imaging modalities have the potential to enable quantitative assessment of vitreous properties such as viscoelasticity in vivo, which could have relevance in assessing the risk of progression of vitreoretinal diseases. In this article, we discuss the current clinical approaches to vitreous biomechanics and review relevant new imaging methods with potential for clinical translation.

## 1. Introduction

The vitreous is a clear hydrogel in the eye that resides in the volume between the lens and the retina. While the optical clarity of the vitreous allows for sharp vision, such clarity has posed challenges to the scientific investigation of vitreous properties in vivo. Ex vivo human and animal studies have characterized the physical and chemical properties of the vitreous in normal aging and pathology, which can lead to blinding conditions like retinal holes, tears, and rhegmatogenous retinal detachments [1,2,3,4].

In particular, the biomechanical properties of the vitreous may be directly affected by vitreoretinal pathology, including vitreous hemorrhage, macular holes, and retinal tears. Prior studies have described the delicate intrinsic structure of the vitreous in detail as consisting of layers and cisterns, with broad vitreoretinal adhesions within the vitreous base anteriorly and at the optic disc posteriorly. More focal vitreoretinal adhesions have been observed along the retinal vessels, macula, and fovea as well [5]. These focal adhesions can lead to pathologic damage by causing traction on the macula, retinal vessels, or of the peripheral retina, which can ultimately lead to permanent vision loss, requiring urgent surgical repair.

The importance of the examination of the vitreous was accelerated by the postulates of Jules Gonin, who first theorized that retinal detachments were the result of retinal breaks, suggested that the vitreoretinal interface plays a pathophysiologic role, and developed the first effective treatment for retinal detachment [6]. As ophthalmic examination became more sophisticated, retina surgeons continued to describe their findings of the vitreoretinal interface and develop improved treatments for retinal detachment [7].

The effects of the biomechanical vitreous properties have been well-described subjectively, clinically, and histologically, but the quantitative physical properties of the vitreous remain challenging to describe in vivo due to its optical clarity, relatively large volume, and ocular motion. Vitreous biomechanics is a broad term that refers to many specific characteristics including dynamic measures like bulk movement, oscillatory movement and damping; static measures like viscosity, elasticity, and density; and response to force including axial stress, shear stress, and torsional stress. These specific parameters, though distinct, are connected in the pathophysiology of vitreoretinal disease.

For example, following an ocular saccade, there is bulk rotational and oscillatory movements of the vitreous. The transmission of the forces within the vitreous in these bulk movements depends on the viscosity and elasticity of the vitreous. The vitreous then undergoes deformation based on its physical stress, which alters the vitreoretinal interface, causing vitreoretinal traction that can ultimately lead to retinal breaks and retinal detachments. We review all methods of assessing vitreous biomechanics relevant in the pathophysiology of vitreoretinal disease, with a particular focus on viscoelasticity. Some additional imaging methodologies that may be indirectly related to biomechanics are discussed as well.

Each of these factors have been studied, but investigations are lacking into quantifying the biomechanical features of the vitreous in vivo using imaging. Additionally, there is a lack of ex vivo correlation to traditional rheological and viscoelastic measurements. Due to the lack of quantitation both in vivo and ex vivo, the correlation of vitreous biomechanics, assessed via imaging, to clinically important pathologies like retinal detachment has not been established in the literature. However, an understanding of such correlation could be helpful in identifying patients at risk of progression of vitreoretinal disease and could impact patient outcomes by leading to earlier intervention and closer monitoring.

Many methods have been used to clinically describe the physical properties of the vitreous including fundus photography, ultrasound, and optical coherence tomography. In recent years, several advanced imaging modalities have been used to further specify vitreous properties such as viscosity. In this article, the clinical changes of the vitreous with normal aging and disease are discussed, followed by a review of clinical and research imaging methods used to quantify these physical properties of the vitreous. The purpose of this review is to summarize the literature on vitreous biomechanical imaging with a focus on viscoelasticity. The limitations of the existing literature are highlighted, and potential future directions are discussed, with the ultimate goal of identifying modalities with the most potential for translation to the clinical environment and improve visual outcomes for patients with vitreoretinal disease.

## 2. Methodology

This narrative review of the literature was conducted using the PubMed database using the following search terms: “vitreous imaging biomechanics,” “vitreous imaging viscosity,” and “vitreous imaging viscoelasticity.” Studies using animal or human vitreous humor in vivo or ex vivo were included. Any study that did not investigate the vitreous humor was excluded, as were studies that investigated symptomatic floaters of the vitreous, myodesopsias, case reports, and vitreoretinal surgical techniques. The search was conducted between 1 November 2025 and 14 November 2025 and yielded 107 studies. After exclusionary criteria were applied, 13 studies met the study criteria and were included in the analysis. Additional studies regarding historical context, vitreous physiology, and clinical features were included and discussed as background.

Identified studies were then divided based on the use of clinically available imaging technology. The mechanism of each imaging modality was reviewed, as well as its specific biomechanical relationship with physiologic parameters, with particular focus on viscoelasticity. The strengths and weaknesses of each approach as a potential translational imaging modality were then summarized and synthesized.

## 3. Clinical Features of the Vitreous

The vitreous is well-formed at birth but undergoes liquefaction, typically over decades, that culminates in the separation of the vitreous from the posterior retina, referred to as posterior vitreous detachment. The liquefaction of the vitreous, also known as syneresis, can be clinically visualized by slit lamp and is known to increase with age [1]. This process occurs in some primates but does not occur in all mammals, based on our limited scientific understanding [8]. Vitreous syneresis leads to posterior vitreous detachment during which the liquefied vitreous prolapses behind the posterior vitreous face. This sequence has been well-described using clinical stages based on slit lamp biomicroscopy [9]. However, there is no objective or comprehensive method for grading or quantifying the degree of vitreous liquefaction, even when it is visibly present.

Recently, clinical imaging methods such as optical coherence tomography (OCT) have been used to characterize stages of normal and pathologic posterior vitreous detachment and vitreomacular adhesion [10]. These staging systems do not account for the entirety of vitreous changes with age and disease but represent a clinically observable outcome related to certain conditions. Moreover, these staging systems describe the current state of the vitreoretinal interface but cannot predict the risk or time of pathologic progression.

Comorbid conditions such as Stickler’s syndrome are associated with inherited collagen mutations and lead to clinical differences in vitreous appearance, resulting in significantly elevated risk of retinal abnormalities including lattice degeneration, retinal tears, and retinal detachment [11]. Prophylactic treatment with cryotherapy or laser retinopexy in such high-risk patients has been shown to be effective in decreasing the frequency of retinal detachment [12]. Patient risk factors such as high myopia, prior retinal detachment, and exam findings such as lattice degeneration and retinal holes are also used to assess the risk of retinal detachment and suggest the need for preventive treatment.

## 4. Clinical Imaging Modalities

Fundus photography was the first objective method used to document vitreoretinal pathology. Traditional color fundus photography captures optical changes in the vitreous like vitreous floaters, including those associated with posterior vitreous detachment, asteroid hyalosis, and vitreous hemorrhage. Other methods like infrared ultra-wide-field confocal scanning laser ophthalmoscopy may more accurately evaluate visually significant floaters and myodesopsia [13]. While these fundus photographic methods accurately and objectively document changes in the vitreous, static photography is incapable of investigating the dynamic mechanical properties of the vitreous, which are most relevant in pathophysiology. Dynamic imaging methods have been explored, such as infrared videography, which has been used to grade the visual impact of vitreous floaters. Although kinetic movement is captured with videography, this alone provides no assessment of the pathologic tissue changes that may be related [14].

Dynamic imaging of the vitreous has also been studied with ultrasound and OCT [5,15]. Ocular echography, or ultrasound, has been used clinically for decades as an adjunct to clinical exam. Many clinical vitreous findings can be obtained with ultrasound including vitreous movement, vitreous attachments, syneresis and posterior vitreous detachment [15]. However, similar to a clinical exam, these findings provide limited quantitative comparisons of vitreous properties like viscosity and density. Some quantification may account for visually significant floaters, but these have not been shown to relate to pathogenic qualities like abnormal vitreoretinal adhesions [16]. Other changes have been captured on ultrasound such as an increase in vitreous echogenicity following intravitreal injections, but the clinical consequence of these changes are unclear [17].

Real-time dynamic ultrasonography can visualize the movement of the vitreous, and some studies have attempted to quantify this movement. One small study measured the angular velocity of the vitreous in response to eye movement [18]. The authors reported moderate reproducibility between graders and found a relationship between the movement characteristics of the vitreous and patient characteristics including age, refractive error, and retinal detachment. Another study used Doppler ultrasonography in the setting of horizontal eye movement and assessed the stiffness of the vitreous based on the degree of movement observed following posterior vitreous detachment [19]. While able to measure vitreous movement, these studies fall short of producing robust quantifiable parameters suitable for larger-scale clinical investigation. Ultrasound elastography, which measures the response of the vitreous in response to physical compression by the examiner, showed a decrease in the elasticity of the posterior vitreous in a study of five patients with posterior vitreous detachment [20]. However, the study was limited to eyes with no vision due to safety concerns on the effect of the study protocol on vision, which limits general application of this technique.

Optical coherence tomography has become routine in the assessment of ophthalmic conditions that affect the posterior pole of the eye. OCT can also be used to describe and stage vitreomacular pathology but is generally limited to regions posterior to the equator. Limited studies have been conducted to quantify changes in the vitreous using OCT. Some work has been performed to quantify the vitreous haze visible on OCT by comparing the vitreous reflectivity to that of the outer retina [21]. The goal in the study, however, was the standardization of uveitis grading and did not focus on the physical properties of the vitreous itself. To improve signal detection of the vitreous, frequency-boosted OCT has been reported to provide additional structural information on the vitreous and vitreomacular pathologies [5,22]. These studies characterized the location and configuration of the vitreous, but they were not able to quantitatively assess the vitreous. OCT has been used to describe to movement of vitreous floaters using the en face near-infrared images of commercial OCT systems [23]. Phase-decorrelation OCT, which is not currently commercially available, measures the complex decorrelation of OCT over time and is related to viscosity in addition to other variable like scattering particle size and temperature [24,25]. This technology has been applied to corneal and lenticular pathology and shows potential for studying the vitreous as well.

Another imaging modality capability of assessing vitreous dynamics is magnetic resonance imaging (MRI). Several studies have investigated the use of MRI in quantifying the age-related changes in the vitreous. The T2 relaxation time of the vitreous ex vivo has been shown to correspond to the degree of liquefaction, changing with aging and in the antero-posterior axis [26]. Nuclear magnetic resonance imaging has produced similar findings [27]. Dynamic MRI, measuring the movements of the vitreous with eye movements in vivo, has captured vitreous deformation in a polyphasic pattern corresponding to the gel and liquid phases of the vitreous in a small number of patients [28]. However, MRI is relatively expensive, time-intensive, and has limited spatial and temporal resolution. The advantages and disadvantages of current clinical methods in examining the vitreous are summarized in Table 1.

## 5. Alternative Ophthalmic Imaging

**Figure 1:**
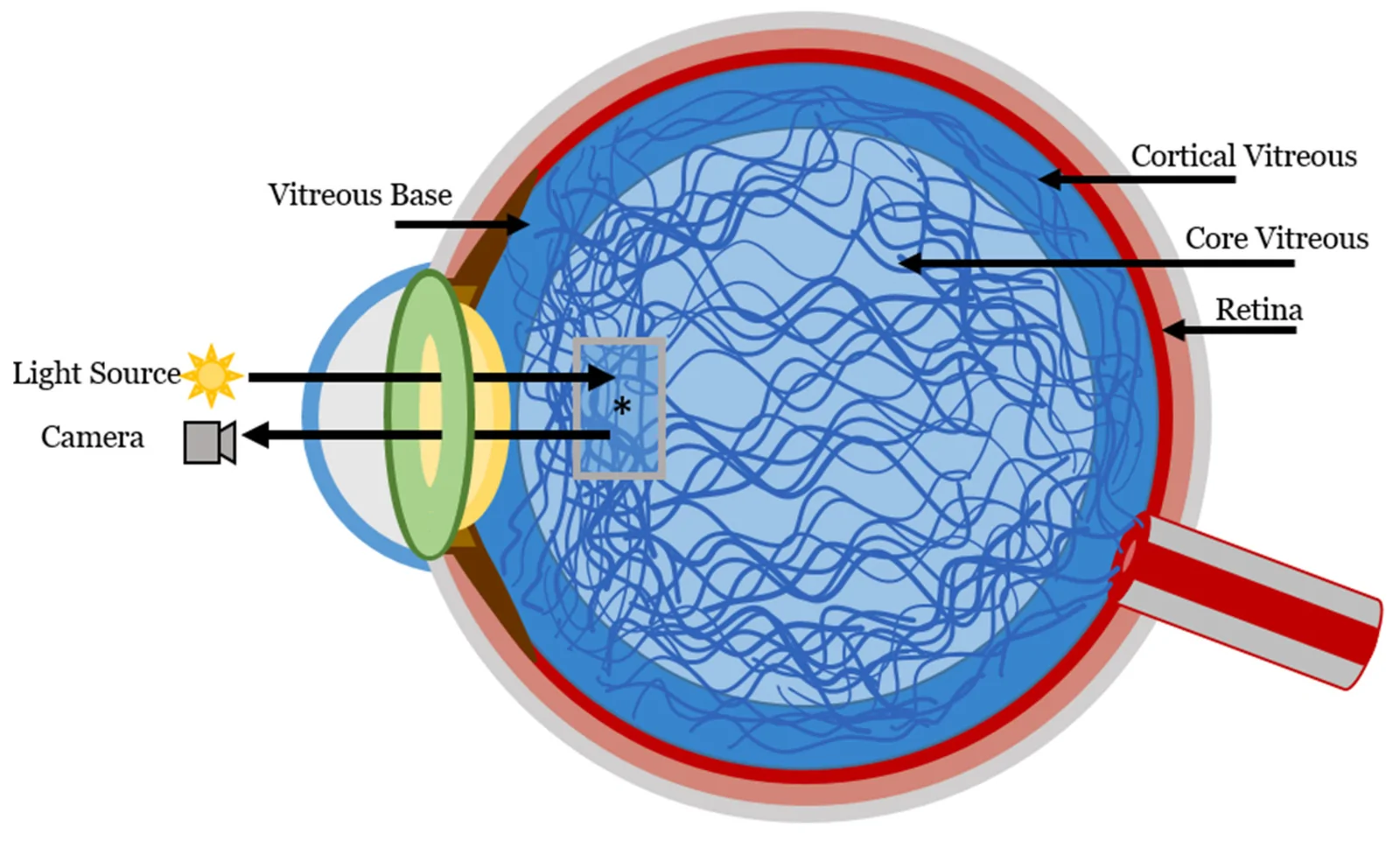
Schematic of vitreous imaging. The vitreous (blue) fills the space between the lens (yellow) and retina (red). Vitreous fibrils are denser in the cortical vitreous and vitreous base (dark blue). The asterisk (*) indicates the arbitrary region of the vitreous undergoing imaging. The light source can be visible light, infrared light, or any other wave including ultrasound. The camera measures reflected light, as in fundus photography, scattered light as in Brillouin spectroscopy, or optical coherence as in optical coherence tomography. Original artwork by Taariq Mohammed, MD.

Several research and pre-clinical imaging modalities have been used to characterize the vitreous, with varying degrees of reported success and reproducibility. Instead of directly measuring reflected light as in traditional fundus photography, the scattering of light is measured. In general, light scatters in multiple ways, and different types of scattering by the vitreous provide different insights into its biochemical composition and physical properties like viscosity and density (Figure 1).

Observation of the motion of the vitreous has been attempted in vivo by measuring the scattering of visible light through human vitreous due to specific head movements. In one study, the vitreous was modeled as a damped oscillator, and this model was used to measure an average damping coefficient in six subjects. It found significant differences between the horizontal and vertical motions of the vitreous, which were attributed to gravitational effects on the vitreous [29]. Dynamic visible light scattering of the vitreous without bulk movement shows some potential to non-invasively quantify vitreous changes spatially due to the Brownian motion of macromolecules like collagen and hyaluronic acid contributing to laser scatter [30].

Other forms of light scattering include Raman and Brillouin scattering. Raman scattering results from inelastic interactions between photons and molecular vibration. Raman spectroscopy provides information on molecular bonds and composition and measures a larger wavelength shift and has a lower amplitude signal. Brillouin spectroscopy provides information on the intrinsic sound waves (or phonons) propagating through a material, measures a smaller wavelength shift and has a larger amplitude signal. Both Raman and Brillouin scattering are inelastic and yield a measurable frequency shift but differ in the information provided.

The information on molecular composition provided by Raman spectroscopy has high potential value for studying the vitreous. Raman spectroscopy has been employed ex vivo to analyze vitreous specimens removed during vitrectomy surgery, for example, quantifying the amount of advanced glycation end-products in proliferative diabetic retinopathy, which may be pathologically important in the progression of disease [31]. Beyond the vitreous, Raman spectroscopy has also been studied for examining human and porcine retina ex vivo [32]. However, due to the relatively low signal strength of Raman scattering, the light intensity required for reliable image processing has limited the study of the human vitreous in vivo due to concerns with phototoxicity.

Brillouin light scattering has been proposed as an optical elastography method, measuring stress and strain for viscoelastic materials, and has been tested with collagen-based hydrogels ex vivo. Brillouin spectroscopy has been used in vivo to characterize corneal and lenticular changes [33]. In high-water-content samples, however, similar to the concentration of the vitreous humor, Brillouin measurements have reduced sensitivity to the viscoelastic properties of the material; in this case, Brillouin linewidth measurements provide a more accurate estimate of material properties than Brillouin frequency shift measures [34,35,36,37]. Other potential advantages of Brillouin spectroscopy for investigating vitreous biomechanics include high spatial resolution in three dimensions and dynamic imaging [38]. Further assessment is needed to determine the effectiveness of Brillouin spectroscopy in characterizing the biomechanical properties of the vitreous in vivo.

Another approach to measuring the dynamic behavior of the vitreous is to assess the diffusion of molecules within the vitreous, which is indirectly related to physical parameters like viscosity and particle size. Labeled probes within a medium are observed for movement, which may be in response to physical stimuli or due to the intrinsic Brownian motion of its particles. For example, labeled probes have been used to study the movement of vitreous analogues during vitrectomy surgery [39]. However, these methods require the use of external probes within the vitreous chamber, which limits clinical use and in vivo investigation.

There are some approaches for assessing diffusion within the vitreous that do not rely on probes. Fluorescence recovery after photobleaching (FRAP) also measures viscosity based on diffusion in the vitreous in ex vivo animal vitreous models [40,41]. A fluorescent probe is mixed and then bleached in a specific area of the vitreous with light. Then, the time for the surrounding fluorescent probe to reconstitute in the bleached area is measured, and this time correlates with the diffusion of the probe and the viscosity of the vitreous. FRAP has not been used in vivo and may be limited by safety considerations given the intensity of blue light required. Another probe-free method is flow-induced cytoplasmic streaming (FLUCS), which utilizes thermoviscous flow. In FLUCS, a focused laser induces local heating that leads to fluid flow. The laser is then oscillated, and the characteristics of the resulting flow have been shown to relate to the bulk rheological properties of viscoelastic materials [42]. FLUCS has not yet been tested in ophthalmic settings.

Adaptive optics can be used to image the hyalocytes in the vitreous cortex and internal limiting membrane [43,44]. This provides a high-resolution view of the cellular component of the vitreous, including capturing hyalocyte movement. Clinical OCT can identify hyalocytes in a similar fashion [45]. However, imaging hyalocytes does not provide direct functional information about the physical properties of the vitreous or vitreoretinal interface but may be used in conjunction with other imaging modalities. Previous studies have looked at hyalocytes in relation to abnormalities of the vitreoretinal interface but again have provided limited quantitative assessment of the vitreous [46]. A summary of selected alternative imaging modalities with potential use for measuring vitreous biomechanics are shown in Table 2.

## 6. Ex Vivo Approaches for Assessing Vitreous Biomechanics

Assessing the imaging-based biomechanical properties of the human vitreous requires ex vivo validation. However, there is no consensus approach for quantifying the physical behavior of the vitreous ex vivo or in vivo. Traditional rheometry poses challenges due to the small volume and complex microstructure of the vitreous, so alternative approaches such as magnetic microrheometry have been employed ex vivo to measure the viscoelastic parameters of the vitreous from donor human eyes. Such studies reported wide variability in measurements, which the authors ascribed to individual differences or possible sampling and handling artifacts. Despite this variability, the study demonstrated significant differences between vitreous regions [47].

Another ex vivo approach is diluting the human vitreous, measuring viscosity, and then extrapolating the properties of the original vitreous. One study examined vitreous changes following cataract extraction comparing a pair of donor eyes frozen 7 h post mortem, where one eye had cataract surgery and the other had a natural phakic lens. They reported that, in the phakic eyes, there was a gradient of decreased viscosity in the posterior vitreous compared to the anterior vitreous but that this gradient reversed following cataract extraction [48]. However, diluting the vitreous may affect the vitreous microstructure, limiting this approach. The same study also measured proteomics and light scattering. Instead of absolute viscosity, a micropipette can be used to measure viscosity relative to a reference compound such as distilled water. A study of human donor eyes found agreement between eyes with this form of measurement; however, there was significant variation between eyes. These differences were again attributed to age-related changes in the vitreous [49].

Several animal models have been employed, although there are significant differences in vitreous composition and behavior even within mammalian models [1]. In rabbits, baseline studies have shown an antero-posterior gradient of vitreous composition [50]. Relative viscosity was calculated in comparison to saline between normal vitreous and vitreous injected with hyaluronidase as a model for vitreous degradation. Overall values were lower in comparison to those of bovine vitreous, with decreased concentrations of hydroxyproline (a surrogate for collagen) and hyaluronic acid. More recent studies have compared human, bovine, and porcine vitreous using rotational rheometers [51]. They have found an age-related decline in viscoelasticity, independent of gender and the presence of diabetes. The authors found that both types of animal vitreous were similar to those in older humans and did not report spatial changes within the vitreous.

## 7. Challenges and Future Directions

There are many challenges in studying the biomechanical properties of the vitreous using imaging, which accordingly offers many potential future research directions. In this narrative review, we summarized the current literature in the field of vitreous biomechanics, with a focus on imaging methods that assess viscoelasticity. While a systematic review of quality and bias would be important, the limitations of the literature as highlighted here precluded a formal systemic review. In addition, there are additional imaging modalities such as magnetic resonance elastography that are used for capturing other important biomechanical features of the vitreous, including vitreoretinal traction and strain, but these were outside the scope of this review.

There are multiple clinical imaging modalities that provide some insight into the vitreous, as summarized in Table 1. When used dynamically, they provide some assessment of vitreous biomechanics including bulk movement and oscillatory behavior but do not or cannot assess other important physical features like viscosity. The alternative imaging modalities, described in Table 2, are able to measure viscoelasticity with a high level of spatial resolution. However, due to a lack of proven efficacy, these imaging modalities are largely available in a research context, which limits clinical availability. Each modality has strengths and weakness, but there some shared challenges in assessing vitreous biomechanics using imaging in general. Selected key studies are summarized in Table 3, including primary findings and limitations.

The largest challenge is the lack of clinical correlation of imaging-based vitreous biomechanical measurements to meaningful patient outcomes, like the risk of development of rhegmatogenous retinal detachment. Such correlation has a biological basis, as demonstrated in collagen disorders such as Stickler syndrome that increase the risk of retinal detachment. Due to the biochemical changes in collagen, there are associated visible clinical changes in the vitreous, and early intervention in patients with Stickler syndrome has been shown to dramatically reduce the lifetime risk of retinal detachment [12]. In this condition, a biochemical change in collagen composition alters the physical behavior of the vitreous in a way that increases the risk of retinal detachment and is responsive to treatment.

Despite biological plausibility, there is a lack of clinical correlation to vitreous imaging due to several factors. Some imaging modalities, such as fluorescence recovery after photo-bleaching and flow-induced cytoplasmic streaming, have not yet been studied in vivo. Safety is a limiting factor in optical methods of assessment due to the potential for retinal phototoxicity. For FDA approval, in vivo imaging methods need to comply with the ocular safety standards set by the American National Standards Institute [52]. These standards account for wavelength, power, and focus and provide safety limits for human imaging.

For imaging modalities that have been studied in vivo, such as ultrasound and structural OCT, the number of patients studied is low, limiting the assessment of reproducibility and accuracy. Low sample size also precludes robust repeatability measurements, accounting for factors such as temperature, intraocular pressure, ocular comorbidities, and eye movement. In addition, no studies followed patients longitudinally, which limits assessment of sensitivity, specificity, and risk for specific vitreoretinal conditions. Because no clinical correlation has yet been found, there has not been any clinical adoption of newer techniques for assessing vitreous biomechanics.

Another limiting factor is the lack of ex vivo quantification of physical vitreous properties. Estimates of human vitreous viscosity from ex vivo models, for example, span a wide range of values, with a high level of intra-eye and inter-eye variability. There is no accepted standard biomechanical ground truth measurement of vitreous biomechanics such as viscoelasticity, which negatively affects the ability to correlate with individual imaging-based measurements and to methodically compare the effectiveness of each imaging modality. Ex vivo validation would further need to consider the anatomy of the vitreous and sampling location, post mortem changes, and differences between the human and animal vitreous.

An optimal method would need to be able to reliably analyze microliters of vitreous to account for the heterogeneity within the vitreous humor. The ex vivo methods discussed in this paper fall short of providing a standardized and reproducible assessment of vitreous viscosity. New methods for assessing viscosity using microliters of fluid have the potential to account for the intrinsic structure of the vitreous, which will more accurately correlate with future translational methods with high spatial resolution. These methods track micro-particles whose movements correlate with viscosity, and, while they have not yet been applied to the vitreous, they are promising future avenues to determine correlation with imaging metrics [53,54].

Although it is difficult to compare imaging modalities directly, there are several important features of a potential translational imaging modality that can be compared. Safety is paramount, in particular with the risk of retinal phototoxicity, and the adoption of several technologies such as fluorescence recovery after photo-bleaching may be limited by safety. Cost and accessibility are important factors as well. Additionally, the optimal translation imaging modality must also have a quantitative output that is directly linked to a measure of viscoelasticity, validated by ex vivo rheological assessment. There is no single imaging modality identified in this review that meets these requirements, but two methods—Brillouin spectroscopy and phase-decorrelation OCT—are the most promising. Both have demonstrated safety in ophthalmic use, have quantitative output that correlates with viscosity, and can be acquired with a high degree of spatial resolution.

Further directions include ex vivo validation of imaging metrics of viscoelasticity, using micro-rheological and micro-viscometric techniques. A potential method for providing ex vivo validation would be to analyze vitreous samples with a translational imaging modality, followed by analysis with microparticle tracking rheology, to determine the degree of correlation. Once validated, the translational imaging technique can then be studied cross-sectionally in humans to identify changes associated with known risk factors for different types of retinal detachment such as myopia and diabetes. Potential confounders will need to be controlled, such as comorbid ocular disease, intraocular pressure and axial length. Then, prospective longitudinal studies will be required to quantify the risk of developing retinal detachment associated with biomechanical vitreous changes identified with imaging. Finally, clinical consensus on the management of these high risk imaging features of the vitreous can be achieved.

## 8. Conclusions

The clinical importance of vitreous biomechanics is evident in a range of vitreoretinal pathologies that are potentially blinding. Despite decades of scientific investigation from bench to bedside, there are limited quantitative methods for assessing the biomechanical properties of the vitreous, especially in the living eye. There are several newer optical methods for investigating the biomechanics of the human vitreous non-invasively, but the relationship of these measurements to clinically meaningful abnormalities is not well-understood. Further insight into the physical behavior of the vitreous may yield improved visual outcomes for patients by identifying treatable pathology earlier.

## Figures and Tables

**Table 1:** Clinical modalities for examining the vitreous humor.

	Advantages	Disadvantages
**Clinical Examination**	No additional cost, time or equipment required Can be dynamic with scleral depression Can examine the entire retina and vitreoretinal interface	No method for quantification Requires time and trained practitioner to perform
**Fundus Photography**	Some methods can assess peripheral vitreoretinal changes Low cost, high accessibility	Color artifacts can occur with vitreous abnormalities Variable assessment of far peripheral retina
**Ultrasound**	Works with media opacities (e.g., corneal scar, cataract) Can show dynamic changes Low cost	Requires time and a trained practitioner, limiting accessibility Limited resolution for small or focal pathology
**OCT**	High-resolution assessment of macular pathology Can easily trend over time Low cost, high accessibility	Very limited for peripheral retinal changes
**MRI**	Whole-eye measurement including objective measurements of globe size Can show dynamic changes	Costly and time-consuming, with limited accessibility for high-resolution imaging Limited spatial and temporal resolution

**Table 2:** Summary of selected alternative imaging modalities with potential use in examining vitreous biomechanics.

Approach	Mechanism	Output	Spatial and Temporal Resolution	Use In Vivo and Clinical Availability	Strengths	Weaknesses
**Brillouin Spectroscopy**	Measures light scattered by intrinsic sound waves	Brillouin shift and linewidth, which are related to Young’s modulus and viscosity respectively	Microns, seconds	Performed in vivo [unpublished data], not clinically available	Potential to directly measure viscoelastic properties High spatial resolution Favorable safety profile for in vivo imaging	Potential limitations in samples with high water content Limited temporal resolution
**Raman Spectroscopy**	Measures light scattered by molecular bond vibrations	Raman shift, which is related to vibrational modes of a molecule	Microns, minutes	Not performed in vivo, not clinically available	Assessment of chemical properties High spatial resolution	Signal strength limited by safety in vivo Does not assess biomechanical properties
**Flow induced cytoplasmic streaming**	Measures light scattered by induced heat flows	Phase angle of stress–strain relationship, a measure of viscoelasticity	Microns, milliseconds	Not performed in vivo, not clinically available	Direct measurement of viscosity Induced signal can be stronger than baseline scattering from Brownian motion	Potential safety concerns in vivo Needs to be coupled with other imaging modality
**Fluorescence recovery after photobleaching**	Measures the duration a photobleached volume takes to reconstitute	Time duration, related to viscosity and diffusion	Microns, seconds	Not performed in vivo, not clinically available	Can directly measure viscosity and diffusion	Safety concerns due to potential for phototoxicity Sensitive to bulk movement
**Adaptive optics**	Compensates for optical aberration using adjustable mirrors to improve image quality	Static image, which is not directly related to biomechanics	Microns, milliseconds	Performed in vivo [39], clinically available	Can image single hyalocytes Favorable safety profile for in vivo imaging	Does not provide assessment of biomechanical properties
**Phase decorrelation OCT**	Measures complex valued optical coherence as it changes over time	Optical coherence phase contrast, which is related to viscoelasticity	Microns, seconds	Not performed in vivo, not clinically available	Potential to directly measure viscoelastic properties High spatial resolution Favorable safety profile for in vivo imaging	Needs reliable scattering elements Affected by scattering particle size

**Table 3:** Summary of selected key trials studying the vitreous in human eyes with quantitative biomechanical metrics.

Authors	Modality	Sample Size	Key Findings	Major Limitations
**Zimmerman [29]**	Direct observation	N = 6	Bulk vitreous rotation can be modeled as damped oscillation	Only assessed one specific type of vitreous movement Multiple observers unable to complete experiment due to incomplete visualization
**Rossi et al. [18]**	Ultrasound velocimetry	N = 12	Age and high myopia were associated with change in vitreous velocity measured with ultrasound	Potential artifact from heterogeneous ultrasound signal from unfixed focal plane; studied only patients with vitreous pathology
**Detorakis et al. [20]**	Ultrasound elastography	N = 5	Ultrasound elastography displayed changes in the anterior and posterior vitreous, corresponding to posterior vitreous detachment	Performed in non-sighted eyes due to safety concerns
**Stein et al. [26]**	Magnetic resonance imaging	N = 20	T2 relaxation time increased with age and within eyes posteriorly, corresponding to increased vitreous liquefaction	Performed in ex vivo eyes, with possibility for post mortem artifacts; limited access to 7 Tesla machines
**Piccirelli et al. [28]**	Dynamic magnetic resonance imaging	N = 19	Polyphasic vitreous movement was observed in some patients, possibly related to posterior vitreous liquefaction	Limited spatial resolution; only assessed one specific type of vitreous movement

## Data Availability

No new data were created or analyzed in this study. Data sharing is not applicable to this article.
